# Diagnostic Performances of Ultrasound-Based Models for Predicting Malignancy in Patients with Adnexal Masses

**DOI:** 10.3390/healthcare11010008

**Published:** 2022-12-20

**Authors:** Clarissa L. Velayo, Kareen N. Reforma, Renee Vina G. Sicam, Michele H. Diwa, Alvin Duke R. Sy, Ourlad Alzeus G. Tantengco

**Affiliations:** 1 Department of Physiology, College of Medicine, University of the Philippines Manila, Manila 1000, Philippines; 2 Department of Obstetrics and Gynecology, University of the Philippines—Philippine General Hospital, Manila 1000, Philippines; 3 Department of Pathology, College of Medicine, University of the Philippines Manila, Manila 1000, Philippines; 4 Department of Epidemiology and Biostatistics, College of Public Health, University of the Philippines Manila, Manila 1000, Philippines; 5 College of Medicine, University of the Philippines Manila, Manila 1100, Philippines; 6 Career Incentive Program, Department of Science and Technology-Science Education Institute, General Santos Avenue, Bicutan, Taguig City 1630, Philippines

**Keywords:** ADNEX, IOTA, ovarian cancer, Philippines, simple rules, CA-125, MIA2G

## Abstract

This study compared the diagnostic performance of different ultrasound-based models in discriminating between benign and malignant ovarian masses in a Filipino population. This was a prospective cohort study in women with findings of an ovarian mass on ultrasound. All included patients underwent a physical examination before level III specialist ultrasonographic and Doppler evaluation using the different International Ovarian Tumor Analysis (IOTA) Group’s risk models. Serum CA-125 and a second-generation multivariate index assay (MIA2G) were also determined for all patients. The ovarian imaging and biomarker results were correlated with the histological findings. A total of 260 patients with completed ultrasound, CA-125, MIA2G, and histopathologic results was included in the study. The presence of papillae with blood flow and irregular cyst walls during the ultrasound were significantly associated with a 20-fold (OR: 20.13, CI: 8.69–46.67, *p* < 0.01) and 10-fold (OR: 10.11, CI: 5.30–19.28, *p* < 0.01) increase in the likelihood of a malignant lesion, respectively. All individual sonologic procedures performed well in discerning malignant and benign ovarian lesions. IOTA-LR1 showed the highest accuracy (82.6%, 95% CI: 77.5–87%) for identifying ovarian cancer. IOTA-ADNEX showed the highest sensitivity (93.3%, 95% CI: 87.2–97.1%) while IOTA-LR2 exhibited the highest specificity (84.4%, 95% CI: 77.3–90%). Among the different serial test combinations, IOTA-LR1 with MIA2G and IOTA-LR2 with MIA2G showed the highest diagnostic accuracy (AUROC = 0.82). This study showed that all individual ultrasound-based models performed well in discerning malignant and benign ovarian lesions, with IOTA-LR1 exhibiting the highest accuracy.

## 1. Introduction

Ovarian cancer was the third most common gynecological cancer with a total of 313,959 new cases of ovarian cancer recorded globally in 2020 [1,2]. It also has the highest mortality (4.2 per 100,000) rate with a total of 207,252 new deaths globally [1]. The highest mortality rate was recorded in Micronesia (7.3 per 100,000), followed by Polynesia (6.6 per 100,000), Central and Eastern Europe (5.6 per 100,000), and Southeast Asia (5.2 per 100,000) [1]. This high mortality rate was attributed to the diagnosis of ovarian cancer at later stages of the disease [3]. The five-year survival rate at these late stages was less than 30.8% while at earlier stages of the disease, the rates ranged from 74.2 to 93.1% [4]. Thus, the development of screening tools for the early detection and management of ovarian cancer would make a significant impact on the prognosis of each patient.

Currently, the standard screening method for ovarian cancer involves traditional ultrasound imaging combined with cancer antigen 125 (CA-125) [5]. Over the past two decades, there has been several ultrasound-based risk prediction systems developed to guide sonographers in classifying ovarian tumors. In the Philippines, obstetrician-gynecologists use these different models including the Risk of Malignancy Index (RMI) and the various models introduced by the International Ovarian Tumor Analysis (IOTA) group. Several local independent reports have individually validated the diagnostic performance of these different ultrasound-based models in screening patients with ovarian masses for ovarian cancer [6,7,8,9]. However, there has been no local comprehensive report to evaluate a comparison of all these different ultrasound-based models. Our work evaluating IOTA-LR2 combined with a multivariate index assay (MIA2G) has previously been reported [10,11]. However, the diagnostic performance of other ultrasound-based models in combination with biochemical markers available in the Philippines such as CA-125 and MIA2G has not been validated. These biomarkers were shown to have a good diagnostic accuracy for ovarian malignancy [10,11,12,13].

Validating these models and screening tools in our setting is of paramount importance in the management of ovarian cancer. Determining the best ultrasound-based models is essential for the correct classification of malignancy risk. Hence, in this research, we endeavored to evaluate the clinical performance of different ultrasound models in preoperative assessment and in discriminating between benign and malignant ovarian masses in a Filipino population. This study also assessed the overall utility of these ultrasound models when combined with CA-125 and MIA2G.

## 2. Materials and Methods

**Figure 1:**
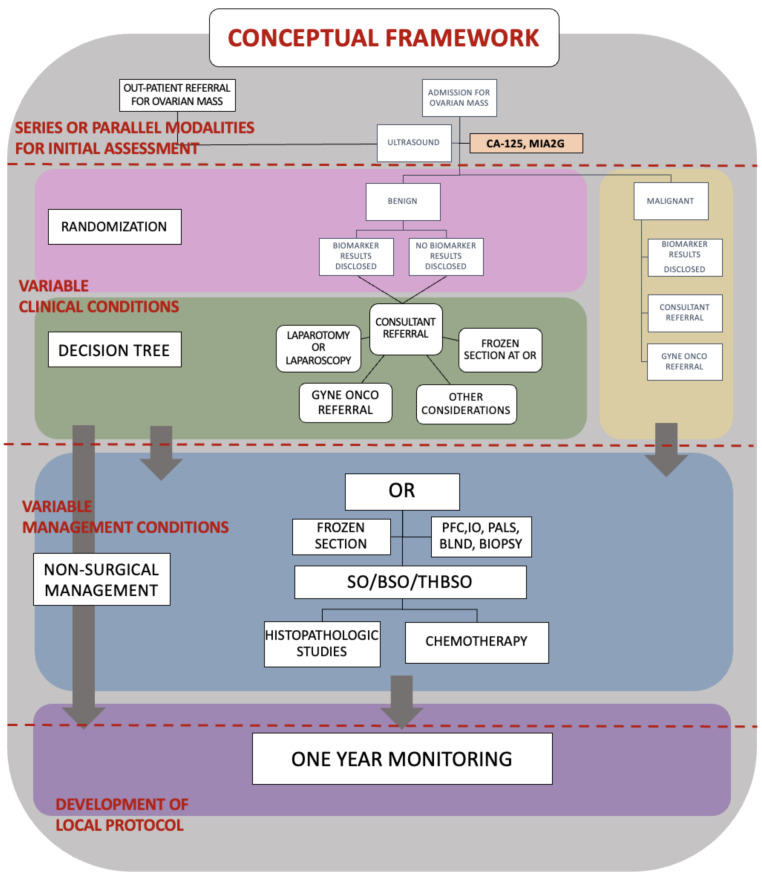
Conceptual framework and methods of the study.

This is a prospective cohort study involving patients with an ovarian mass in the University of the Philippines-Manila and Philippine General Hospital (Figure 1). Our research was approved by the University of the Philippines Manila Research Ethics Board (UPMREB) under UPMREB Code 2017-170-01 and was registered in the Philippine Health Research Registry (PHRR) managed by the Philippine Council for Health and Research Development under Registration ID PHRR180614-001843. Only individuals who met the following inclusion criteria were asked to participate: non-pregnant female, over 18 years of age at recruitment, diagnosed with an ovarian mass, considered for surgery, had not been previously evaluated by a gynecologic-oncologist, and had not been diagnosed with cancer in the past five years. Patients were excluded if they had mental disabilities, severe co-morbid conditions, or were found to be pregnant during initial recruitment.

### 2.1. Data Collection

Each patient underwent a physical examination and medical history interview prior to level III specialist ultrasonographic and Doppler evaluation upon voluntary written consent. All ultrasound tests were conducted and reviewed by a Level III obstetrician-gynecologist trained in ultrasound (KNR). All patients were assessed using different ultrasound-based models and underwent testing for serum CA-125 and MIA2G.

### 2.2. IOTA-LR1 and IOTA-LR2

The probability of malignancy within an adnexal mass was estimated by using the IOTA logistic regression models LR1 and LR2. Twelve variables were used for the LR1 calculation: (1) personal history of ovarian cancer (yes  =  1, no  =  0); (2) current use of hormonal therapy (yes  =  1, no  =  0); (3) age of the patient (years); (4) maximum diameter of lesion (mm); (5) evidence of pain during the examination of the mass (yes  =  1, no  =  0); (6) presence of ascites (yes  =  1, no  =  0); (7) presence of blood flow within a solid papillary projection (yes  =  1, no  =  0); (8) purely solid tumor (yes  =  1, no  =  0); (9) maximum diameter of largest solid component (expressed in mm, but with no increase >50 mm); (10) irregular internal cyst walls (yes  =  1, no  =  0); (11) presence of acoustic shadows (yes  =  1, no  =  0); and (12) color score (1–4, where 1 is no flow and 4 is maximum flow).

The International Ovarian Tumor Analysis (IOTA)—Logistic Regression 2 (LR2) model was used to stratify patients into either a high risk (HR) or a low risk (LR) group. The sonographic parameters used were the presence of ascites, the presence of papillations with detectable blood flow, irregular cyst walls, the presence of acoustic shadows, age, and the maximum diameter of the largest solid component. For this study, patients were classified as high risk if their IOTA-LR2 score was ≥10%.

### 2.3. IOTA-ADNEX

The ADNEX model is freely accessible online at https://www.iotagroup.org/adnexmodel/ (accessed on 8 October 2017) and can be downloaded for use in portable applications. The following predictors were included in the ADNEX model: age (years) of the patient at examination, referral center for gynecologic oncology, maximal diameter of the lesion (mm), maximal diameter of the largest solid part (mm), presence of more than ten locules, number of papillary projections (0, 1, 2, 3, and >3), and the presence of acoustic shadows, ascites, and serum CA-125 level (U/mL).

### 2.4. IOTA Simple Rules

IOTA SR included ten predictors divided into benign features and malignant features. The benign features included the presence of a unilocular cyst, the presence of solid components <7 mm in diameter, presence of acoustic shadows, smooth multilocular tumor with the largest diameter <100 mm, and no detectable color Doppler signal. The malignant features were the presence of an irregular solid tumor, ascites, at least four papillary structures, an irregular multilocular mass > 100 mm in diameter, and a strong color Doppler signal. If one or more malignant features was present but the benign feature was absent, the mass was considered malignant. However, if a mass had one or more B features but no malignant features, it was considered benign.

### 2.5. MIA2G Test

A second-generation multivariate index assay (MIA2G) was performed for all patients. MIA2G (OVERA ^®^) combined the levels of five protein biomarkers: apolipoprotein A1 (APOA1), human epididymis protein 4 (HE4), cancer antigen-125 (CA-125), follicle stimulating hormone (FSH), and transferrin (TRF) along with the woman’s menopausal status to generate a numerical risk score between 0.0 and 10.0 using a proprietary algorithm (OvaCalc^®^ software, Aspira Women’s Health, Austin, Texas, USA). A cutoff score of 5.0 conferred a high risk of malignancy.

### 2.6. Reference Standard

The histopathologic diagnosis served as the “gold” standard or variable that represented the “true” presence of disease. The histopathology review was conducted by a pathologist (MHD). The pathological tumor types followed the World Health Organization’s categorization. The stages of malignancy were based on the International Federation of Gynecology and Obstetrics criteria.

### 2.7. Statistical Analysis

The data used for the current study were derived from a larger randomized cohort study involving 379 women with ovarian lesions seen in a tertiary level institution in the Philippines. For the objective of this study, the minimum sample size was 252 women with a preferable distribution of 84 positive and 168 negative cases. This was computed based on an alpha of 0.05, power of 0.80, a hypothesized difference in the area under the curve between serial MIA2G and IOTA-LR2; parallel MIA2G and IOTA-LR2 of about 0.10, a correlation of the biomarker in the positive and negative group at 0.72 and 0.25 respectively; and a ratio of the sample size in negative/positive groups set at 2:1 aligned with the planned randomized cohort.

After the data were extracted by the investigator from the patient charts, all the information was manually entered into an electronic spreadsheet file and subsequent data processing and analysis were carried out using the Stata 13 software. Descriptive statistics such as the mean, median, standard deviation, and range were used to describe the actual age in years, while the frequency and percentage were used for the categorical variables such as pathological diagnosis, stage, sonographic findings, and risk stratification methods. The sensitivity, specificity, positive and negative predictive values, and correct classification rate in classifying benign or malignant lesions were computed with their interval estimates included. The high prevalence of malignant subjects due to the study institution being a tertiary referral center necessitated an adjustment of the prevalence to a lower one agreed at 10% when computing the predictive values.

In order to assess the exposure status of the individuals, all women included in the study were examined using transvaginal and/or transabdominal ultrasound by a single sonographer with Level III training. By considering relevant clinical and sonologic features, the likelihood of benign and malignant tumors was calculated. According to the results of the IOTA group’s recent studies, a cutoff risk set at 20% was related to the best balance between the ADNEX model’s sensitivity and specificity [14], while the cutoff for LR1 and LR2 was set at 0.10 based on recent studies [15].

In addition, the different diagnostic ultrasound criteria were measured upon parallel testing between the multivariate index assay (MIA2G) and IOTA-LR2. Similarly for serial testing, these diagnostic criteria were measured with the imaging-based procedures performed first, followed by select biomarkers such as CA-125 and MIA2G. For the biomarkers such as CA-125, standard recommended cutoff values of 35 and 67 U/mL among premenopausal and postmenopausal women, respectively, were used [16]; cutoff values of 70 and 140 pmol/L were used among premenopausal and postmenopausal women, respectively, for the HE4, and the MIA2G used a cutoff score for malignancy risk at 5.0 and greater. These measurements were conducted upon study recruitment in order to avoid confounding due to medical management prior to surgery.

In assessing the outcome, histopathologic diagnosis of the surgical specimen was used as the reference standard for a definite diagnosis of the ovarian masses. Borderline ovarian tumors were grouped with malignant ovarian tumors. Only women with histopathologic findings were included in the current report.

## 3. Results

**Figure 2:**
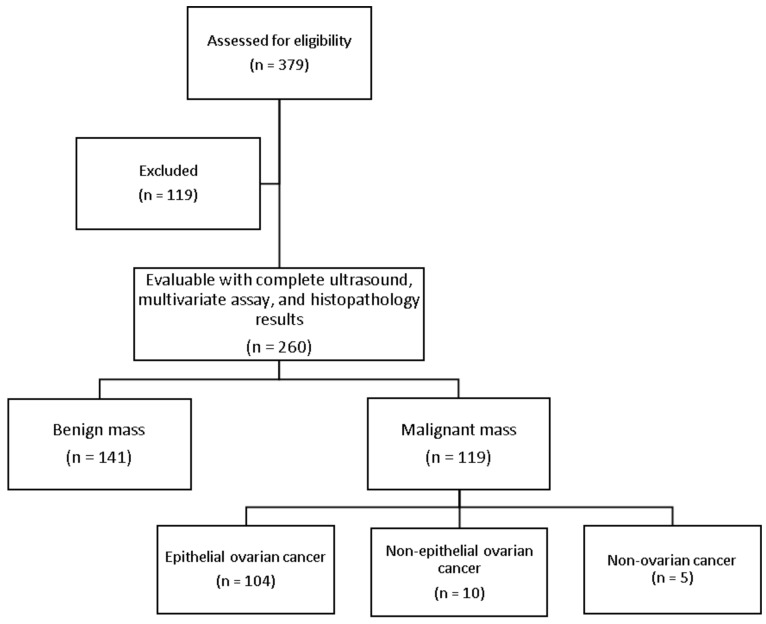
Flowchart showing the final sample size of the participants for this study.

The study recruited 379 patients with ovarian tumors. Based on the inclusion and exclusion criteria, a total of 260 patients with completed ultrasound, CA-125, MIA2G, and histopathologic results were included in the study (Figure 2).

Table 1 shows the clinical and pathologic features of the patients. There were 141 (54.23%) patients with benign ovarian tumors and 119 (45.77%) patients with malignant tumors. Epithelial ovarian cancer (EOC) accounted for 87.39% of the patients with ovarian cancer. Among the EOC patients, the most common histologic type was mucinous (45/104, 43.27%), followed by serous (33/104, 31.73%).

Table 2 shows that the presence of papillae with blood flow and irregular cyst walls during the ultrasound was significantly associated with a 20-fold (OR: 20.13, CI: 8.69–46.67, *p* < 0.01) and 10-fold (OR: 10.11, CI: 5.30–19.28, *p* < 0.01) increase in the likelihood of a malignant lesion, respectively. These findings were supported by an acceptable degree of accuracy, 75% (95% CI: 69.3–80.1%) for the presence of papillae with blood flow, and 73.5% (95% CI: 67.7–78.7%) for irregular cyst walls.

We first assessed the diagnostic performance of the different ultrasound-based models when used alone. Based on Table 3, all individual sonologic procedures performed well in discerning the malignant and benign ovarian lesions. IOTA-LR1 showed the highest accuracy (82.6%, 95% CI: 77.5–87%) for identifying ovarian cancer. IOTA-ADNEX showed the highest sensitivity (93.3%, 95% CI: 87.2–97.1%) while IOTA-LR2 exhibited the highest specificity (84.4%, 95% CI: 77.3–90%).

We then tested the diagnostic performance of the different ultrasound-based models when combined with CA-125 and MIA2G in parallel (Table 4) and in serial testing (Table 5). CA-125 performed better than MIA2G when combined as a parallel procedure with any of the known ultrasound risk scoring for the detecting of ovarian lesions (*p* < 0.01) (Table 4). However, as a serial procedure, MIA2G combined with sonologic risk scoring performed better than serial use of CA-125, as seen in Table 5. Moreover, there was no sufficient evidence to suggest that MIA2G performed better than CA-125 as a serial procedure with remaining ultrasound risk scoring methods for the detection of ovarian lesions (*p* > 0.05). Among the different serial tests, IOTA-LR1 with MIA2G and IOTA-LR2 with MIA2G showed the highest diagnostic accuracy (AUROC = 0.82).

The researchers also compared the accuracy of the different sonologic methods and the use of biomarkers with the manner of performing them either as a serial or parallel approach, as presented in Table 4 and Table 5, respectively. It can be noted that all ultrasound methods performed serially with MIA2G had a significantly higher accuracy (and AUROC) than when parallel testing was performed (*p* < 0.05). On the other hand, serial performance using CA-125 and ultrasound only performed better than the parallel approach when IOTA-LR2 (*p*: 0.05) and IOTA-ADNEX were used (*p*: 0.05). There was no sufficient evidence to suggest that serial performance was better than parallel when CA-125 was combined with IOTA-LR1 and Simple Rules (*p* > 0.05). However, compared to ultrasound-based models alone, adding CA-125 or MIA2G as serial or parallel tests did not improve the diagnostic performance.

## 4. Discussion

Our study emphasized the racial and ethnic differences in the epidemiology of ovarian cancer. There was a 45.77% prevalence of ovarian cancer among women presenting with ovarian masses. From these, EOC comprised 87.39% of the patients with malignant tumors, which was consistent with previous reports that EOC accounts for almost 95% of all ovarian malignancies [17,18,19]. Earlier studies from other countries have shown a predominance of the serous type of ovarian cancer [20,21,22]. However, mucinous types were the most common histologic subtypes in our population. In comparison, a study by Peres et al. reported that high grade serous ovarian cancer was more common among non-Hispanic White, Hispanic, and African-Americans, while clear cell EOC was more common among Asian/Pacific Islanders [23]. Overall, these contrasting findings further stress the importance of conducting studies for ovarian cancer in different countries to fully understand the epidemiology of the disease. This information can be used to develop evidence-based and locally applicable diagnostic and therapeutic strategies for ovarian cancer control, prevention, and management.

Previous studies have also shown that vascularized tissue, thick septations, and papillary projections were the most important and consistent ultrasound and Doppler predictors of ovarian malignancy [24,25]. Upon the analysis of our study population, the presence of papillae with blood flow and irregular cyst walls were significantly associated with a 20-fold and 10-fold increase in the likelihood of a malignant lesion. Moreover, previous studies have reported the presence of ascites during preoperative assessment as highly predictive of ovarian malignancy in women with a pelvic mass [26,27], but our findings showed that ascites and the presence of acoustic shadowing had the lowest diagnostic accuracy. It has long been established that ascites occurs more frequently in patients with advanced-stage ovarian cancer [28], so it is more likely that the higher proportion of early-stage ovarian cancer patients in our study may have resulted in the lower diagnostic accuracy of this particular ultrasound descriptor.

In our sample population, IOTA-LR1 had the highest diagnostic accuracy (82.6%), followed by IOTA-LR2 (82.3%). Both of their diagnostic performances were comparable to the results of previous studies conducted in the Philippines and in Singapore [9,29]. Significantly, the sensitivity and specificity of these tests, however, were lower in Southeast Asian populations including that of our study compared to previous research conducted in Europe. The latter studies reported a sensitivity of 93.7% for LR1 and 90.2% for LR2 [30,31,32]. Undoubtedly, the diagnostic accuracy of ultrasound-based models will rely heavily on the experience of the sonographer [30,31], thus, to ensure the quality of our findings, all participants in our research were examined by a single level III sonographer thoroughly familiar with the different ultrasound-based models developed by IOTA. Therefore, the lower sensitivity of LR1 and LR2 seen here may be due to the differences in the epidemiology of ovarian cancer (i.e., the predominance of mucinous histologic subtypes and early-stage ovarian cancer patients).

Combining ultrasound-based models with CA-125 and MIA2G in parallel and serial testing had a comparable diagnostic performance with ultrasound-based models alone. This result showed the usefulness of different ultrasound-based models practiced by level III sonographers as stand-alone tests in predicting ovarian malignancy. However, biochemical markers such as CA-125 and MIA2G may be more useful in settings where level III sonographers are not available. These may also be useful for patients with inconclusive determination of malignancy risk by ultrasound features alone. In this study, ultrasound methods performed serially with either MIA2G or CA-125 had significantly higher accuracy (and AUROC) than when parallel testing was performed. Hence, we recommend the use of serial testing for our population.

Our study had several limitations. First, the research was conducted in a single tertiary center with complete diagnostic and surgical capabilities. Second, the level of ultrasonography expertise in this study was high (level III). Thus, the results of this study may not apply when the test is performed by less experienced sonographers or in other areas of the Philippines, where the majority only have access to lower-level health care centers. Nevertheless, this study had some strengths. It is the largest local study validating the applicability of different ultrasound-based models in Filipino women with ovarian cancer. Even as ultrasound-models are expected to perform differently in variable centers and among diverse populations, our study was able to present the usefulness of IOTA-based sonologic risk scoring in predicting ovarian cancer in Filipino women as well as the diagnostic performance of ultrasound-based models when combined with biochemical markers such as CA-125 and MIA2G. Finally, biomarkers measured from all patients were analyzed using the same assay kit from the same laboratory, which consequently allowed us to avoid any resulting potential bias with regards to this in our findings.

## 5. Conclusions

This study showed that all individual ultrasound-based models performed well in discerning malignant and benign ovarian lesions. IOTA-LR1 showed the highest diagnostic accuracy. While this study did not show improvement in diagnostic accuracy when ultrasound-based models were combined with either CA-125 or MIA2G, these biomarkers may still be useful in settings where level III sonographers are not available or when the ultrasound findings are inconclusive. However, further multicenter studies are still needed to verify this assumption.

## Figures and Tables

**Table 1:** Demographic characteristics and pathologic results for the evaluable subjects.

Characteristics	All Evaluable Women		
	Overall	Pre-Menopausal	Menopausal
Number	260	169	91
Age in years			
Mean ± SD	44.21 ± 13.00	36.86 ± 9.40	57.73 ± 5.86
Median	45	38	58
Range	18 to 78	18 to 52	42 to 78
Number of pregnancies			
None	71 (27.31%)	58 (34.32%)	13 (14.29%)
1	36 (13.85%)	31 (18.34%)	5 (5.49%)
2	34 (13.08%)	24 (14.20%)	10 (10.99%)
3	22 (8.46%)	13 (7.69%)	9 (9.89%)
≥4	85 (32.69%)	36 (21.30%)	49 (53.85%)
Not specified	12 (4.62%)	7 (4.14%)	5 (5.49%)
Pathologic diagnosis, n (%)			
Benign conditions	141 (54.23%)	104 (61.54%)	37 (40.66%)
Malignant conditions	119 (45.77%)	65 (38.46%)	54 (59.34%)
Epithelial ovarian cancer	104 (40%)	55 (32.54%)	49 (53.85%)
Serous	33 (12.69)	14 (8.28%)	19 (20.88%)
Mucinous	46 (17.69%)	30 (17.75%)	16 (17.58%)
Endometrioid	3 (1.15%)	1 (0.59%)	2 (2.20%)
Clear cell	8 (3.08%)	5 (2.96%)	3 (3.30%)
Seromucinous	2 (0.77%)	-	2 (2.20%)
Brenner	1 (0.38%)	-	1 (1.10%)
Mesonephric-like adenocarcinoma	2 (0.77%)	1 (0.59%)	1 (1.10%)
Mixed	8 (3.08%)	3 (1.78%)	5 (5.49%)
Metastasis (ovarian epithelial in origin)	1 (0.38%)	1 (0.59%)	-
Non-epithelial ovarian	10 (3.85%)	7 (4.14%)	3 (3.30%)
Sex cord stromal	4 (1.54%)	3 (1.78%)	1 (1.10%)
Germ cell	3 (1.15%)	3 (1.78%)	-
Other	3 (1.15%)	1 (0.59%)	2 (2.20%)
Non-ovarian cancer	5 (1.92%)	3 (1.78%)	2 (2.20%)
Stage, n (%)	119	65	54
Low malignant potential	26 (21.85%)	15 (23.08%)	11 (20.37%)
I	50 (42.02%)	32 (49.23%)	18 (33.33%)
II	4 (3.36%)	1 (1.54%)	3 (5.56%)
III	21 (17.65%)	7 (10.77%)	14 (25.93%)
IV	10 (8.40%)	4 (6.15%)	6 (11.11%)

**Table 2:** The presence of papillae with blood flow and irregular cyst walls during the ultrasound.

Descriptors	Malignant	Benign	Accuracy
Ascites			
Present	34	10	63.5% (57.3–69.3%)
Absent	85	131	
Solid components			
Present	34	10	63.5% (57.3–69.3%)
Absent	85	131	
Papillae with blood flow			
Present	61	7	75% (69.3–80.1%)
Absent	58	134	
Irregular cyst walls			
Present	65	15	73.5% (67.7–78.7%)
Absent	54	126	
Acoustic shadows			
Present	2	28	44.2% (38.1–50.5%)
Absent	117	113	
Moderate to Strong flow			
Present	49	11	68.5% (62.4–74.1%)
Absent	70	127	

**Table 3:** Performance of imaging in the identification of ovarian cancer.

Criteria	LR1	LR2	ADNEX	Simple Rules
Number	259	260	260	260
Sensitivity				
%	87.4%	79.8%	93.3%	85.7%
95% CI	80.1–92.8%	71.5–86.6%	87.2–97.1%	78.1–91.5%
Specificity				
%	78.6%	84.4%	62.4%	70.2%
95% CI	70.8–85.1%	77.3–90%	53.9–70.4%	61.9–77.6%
Positive PV				
%	77.6%	81.2%	67.7%	70.8%
95% CI	69.6–84.4%	72.9–87.8%	59.9–74.8%	62.7–78.1%
Negative PV				
%	88%	83.2%	91.7%	85.3%
95% CI	81.0–93.1%	76.1–88.9%	84.2–96.3%	77.6–91.2%
Accuracy				
%	82.6%	82.3%	76.5%	77.3%
n/N	214/259	214/260	199/260	201/260
95% CI	77.5–87%	77.1–86.7%	70.9–81.6%	71.7–82.3%
AUROC	0.83	0.82	0.78	0.78
95% CI	0.78–0.88	0.77–0.87	0.73–0.82	0.73–0.83

**Table 4:** Performance of parallel testing with biomarker assays and imaging in the prediction of ovarian malignancy.

Criteria	LR1		LR2		ADNEX		Simple Rules	
	MIA2G	CA-125	MIA2G	CA-125	MIA2G	CA-125	MIA2G	CA-125
Sensitivity								
%	95.8%	91.6%	94.1%	86.6%	97.5%	94.1%	96.6%	93.3%
95% CI	90.5–98.6%	85.1–95.9%	88.3–97.6%	79.1–92.1%	92.8–99.5%	88.3–97.6%	91.6–99.1%	87.2–97.1%
Specificity								
%	40%	58.6%	40.4%	62.4%	34%	50.4%	34%	52.5%
95% CI	31.8–48.6%	49.9–66.8%	32.3–49%	53.9–70.4%	26.3–42.5%	41.8–58.9%	26.3–42.5%	43.9–60.9%
Positive PV								
%	57.6%	65.3%	57.1%	66%	55.5%	61.5%	55.3%	62.4%
95% CI	50.4–64.6%	57.5–72.5%	49.9–64.2%	58–73.4%	48.5–62.4%	54.1- 68.6%	48.3–62.2%	54.8–69.5%
Negative PV								
%	91.8%	89.1%	89.1%	84.6%	94.1%	91%	92.3%	90.2%
95% CI	81.9–97.3%	80.9–94.7%	78.8–95.5%	76.2–90.9%	83.8–98.8%	82.4–96.3%	81.5–97.9%	81.7–95.7%
Accuracy								
%	65.6%	73.7%	65%	73.5%	63.1%	70.4%	62.7%	71.2%
n/N	170/259	191/259	169/260	191/260	164/260	183/260	163/260	185/260
95% CI	59.5–71.4%	67.9–79%	58.9–70.8%	67.7–78.7%	56.9–69%	64.4–75.9%	56.5–68.6%	65.2–76.6%
AUROC	0.68	0.75	0.67	0.74	0.66	0.72	0.65	0.73
95% CI	0.63–0.72	0.70–0.80	0.63–0.72	0.69–0.80	0.62–0.70	0.68–0.77	0.61–0.70	0.68–0.78

**Table 5:** Performance of serial testing with biomarker assays and imaging in the identification of ovarian malignancy.

Criteria	LR1		LR2		ADNEX		Simple Rules	
	MIA2G	CA-125	MIA2G	CA-125	MIA2G	CA-125	MIA2G	CA-125
Sensitivity								
%	84%	73.9%	78.2%	71.4%	88.2%	77.3%	81.5%	70.6%
95% CI	76.2–90.1%	65.1–81.6%	69.6–85.2%	62.4–79.3%	81–93.4%	68.7–84.5%	73.4–88%	61.5–78.6%
Specificity								
%	80.7%	86.4%	85.8%	88.7%	70.2%	78.7%	78%	84.4%
95% CI	73.2–86.9%	79.6–91.6%	78.9–91.1%	82.2–93.4%	61.9–77.6%	71–85.2%	70.3–84.5%	77.3–90%
Positive PV								
%	78.7%	82.2%	82.3%	84.2%	71.4%	75.4%	75.8%	79.2%
95% CI	70.6–85.5%	73.7–89%	74–88.8%	75.6–90.7%	63.4–78.6%	66.8–82.8%	67.4–82.9%	70.3–86.5%
Negative PV								
%	85.6%	79.6%	82.3%	78.6%	87.6%	80.4%	83.3%	77.3%
95% CI	78.4–91.1%	72.3–85.7%	75.2–88.1%	71.4–84.7%	80.1–93.1%	72.8–86.7%	75.9–89.3%	69.8–83.6%
Accuracy								
%	82.2%	80.7%	82.3%	80.8%	78.5%	78.1%	79.6%	78.1%
n/N	213/259	209/259	214/260	210/260	204/260	203/260	207/260	203/260
95% CI	77–86.7%	75.4–85.3%	77.1–86.7%	75.4–85.4%	73–83.3%	72.5–83%	74.2–84.3%	72.5–83%
AUROC	0.82	0.80	0.82	0.80	0.79	0.78	0.80	0.77
95% CI	0.78–0.87	0.75–0.85	0.77–0.87	0.75–0.85	0.74–0.84	0.73–0.83	0.75–0.85	0.72–0.83

## Data Availability

The data that support the findings of this study are available in this article.
